# Advances on minimally invasive approach for benign total hysterectomy: a systematic review

**DOI:** 10.12688/f1000research.11523.1

**Published:** 2017-08-01

**Authors:** Marina de Paula Andres, Giuliano Moysés Borrelli, Mauricio Simões Abrão

**Affiliations:** 1 Endometriosis Section, Gynecology Division, Faculdade de Medicina, Hospital das Clinicas HCFMUSP, Universidade de Sao Paulo, São Paulo, Brazil

**Keywords:** Hysterectomy, Uterine benign diseases, Uterine myoma

## Abstract

Hysterectomy is one of the most commonly performed gynecologic surgeries, mainly for uterine myomas, abnormal uterine bleeding, and prolapses. It can be performed through several routes, each of which has its advantages and disadvantages. We conducted this systematic review to evaluate recent advances in surgical outcomes of benign total hysterectomies by any route: vaginal (VH), laparoscopic (LH), laparoscopically assisted vaginal (LAVH), single-port (SP), and robotic-assisted laparoscopy (RH). The search was applied to the PubMed electronic database by using keywords “hysterectomy” and “uterine benign disease”, “adenomyosis”, and “myoma”. Prospective and randomized trials of the last 3 years were included. Nine studies were selected and showed that VH was superior to LH, LAVH, and RH in terms of hospital stay and operation time and had the same complication rate and lower costs. SP hysterectomy had no clear advantages over VH or conventional LH.

## Introduction

Hysterectomy is one of the most commonly performed gynecologic surgeries, mostly for uterine myomas, abnormal uterine bleeding, and prolapses. It can be performed through several routes: abdominal, vaginal, laparoscopic, and—more recently—robotic-assisted laparoscopy ^1^. Advantages in using minimally invasive techniques include a decrease in pain, a short hospital stay, decreased infection and blood loss, and better cosmetics. However, a significant increase in cost is observed when using devices such as robotic-assisted laparoscopy or conventional laparoscopy in comparison with vaginal or abdominal hysterectomy ^2, 3^.

Several factors may influence the surgeon’s choice of approach to hysterectomy. Nulliparity, previous surgeries, and suspicion of endometriosis, for example, are usually related to an abdominal or laparoscopic approach, whereas multiparity and uterus with small size and descent are often associated with the vaginal approach. Also, the surgeon’s experience and skills will largely determine the surgical approach to hysterectomy.

Because there are several approaches to hysterectomies, technological innovations are often incorporated into the arsenal for surgical treatments. As the last review performed on this topic was in 2014, we sought to conduct this review to evaluate recent advances in surgical treatment, considering the different approaches and outcomes for hysterectomies performed due to benign pathologies, which constitute the majority of indications.

## Methods

### Search strategy

A thorough search of PubMed/MEDLINE was conducted on the basis of the PRISMA (Preferred Reporting Items for Systematic Reviews and Meta-Analyses) statement ^4^. We used the search terms “hysterectomy” and “uterine benign disease”, “adenomyosis”, and “myoma” as keywords to recover all possible publications on this topic at the PubMed database. Strategies for our electronic search at the Medical Subject Headings (MeSH) database were the following combined MeSH terms with details: (((benign[All Fields] AND (“uterine diseases”[MeSH Terms] OR (“uterine”[All Fields] AND “diseases”[All Fields]) OR “uterine diseases”[All Fields] OR (“uterine”[All Fields] AND “disease”[All Fields]) OR “uterine disease”[All Fields])) OR (“myoma”[MeSH Terms] OR “myoma”[All Fields])) OR (“adenomyosis”[MeSH Terms] OR “adenomyosis”[All Fields])) AND (“hysterectomy”[MeSH Terms] OR “hysterectomy”[All Fields]). Two authors (MPA and GMB) independently assessed the studies for inclusion/exclusion, risk of bias, and extracted data. References of articles were also manually reviewed for other relevant articles.

### Selection criteria and eligibility

We included all comparative trials (randomized clinical trials, case-control studies, and prospective cohorts) that assessed outcomes of total hysterectomy by any route: totally vaginal hysterectomy (VH), laparoscopic hysterectomy (LH), laparoscopically assisted VH (LAVH), single-port hysterectomy (SP-H), and robotic hysterectomy (RH). Outcomes were the following: operation time, estimated blood loss, early post-operative pain symptoms (evaluated by visual analogic scale, or VAS), number of post-operative days in hospital, uterine weight, concomitant procedures, and minor complications (fever) and major complications (wound or vaginal cuff infection, blood transfusion, urinary tract injury, bowel injury, or need of re-operation).

### Data extraction

One author abstracted the data into tables (MPA) and a second author (GMB) independently confirmed the accuracy of the data abstraction.

## Results

### Study selection

**Figure 1.**
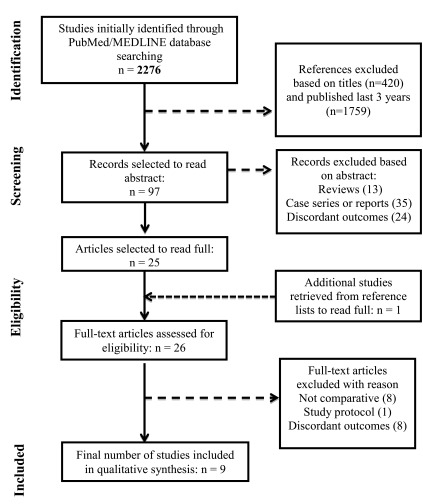
Flow diagram showing the selection of articles for systematic review.

Using the search strategy, we identified 2,276 non-duplicate titles, reviewed 97 abstracts and references, and obtained 26 full-text articles for consideration. We excluded 17 of these full-text articles for the following reasons: not comparative studies, study protocols, and articles that did not evaluate the outcomes described ( Figure 1).

### Characteristics of studies

Nine studies were eligible for inclusion (five randomized clinical trials and four case-control studies) ^3, 5– 12^. Four of them evaluated VH ^3, 6– 8^; four evaluated SP-H ^9– 12^ and one was of these was robotic-assisted ^12^; and six included LH ^3, 5, 7– 9, 11^, two of them with the LAVH approach ^5, 8^ ( Table 1). Studies excluded suspicious or known malignancy, simultaneous need for prolapse surgery, known extensive intra-abdominal adhesion, and uterine volume at more than 16 to 18 weeks of gestation.

**Table 1.** Summary of abstracted data of included studies on hysterectomy.

Reference	Year	Study design	Intervention	Number
Sesti *et al*. ^5^	2014	RCT	VH versus LH VH versus LAVH	108
Chen *et al*. ^6^	2015	RCT	Nerve sparing VH versus conventional VH	300
Lönnerfors *et al*. ^3^	2015	RCT	VH versus LH	122
Bogani *et al*. ^7^	2015	Prospective cohort	VH versus LH	80
Kovachev *et al*. ^8^	2016	Prospective cohort	VH versus LAVH	187
Kim *et al*. ^9^	2015	RCT	SP-LH versus LH	243
Song *et al*. ^10^	2015	RCT	SP-LH versus SP-LAVH	76
Angioni *et al*. ^11^	2015	Case-control	SP-LH versus LH	61
Paek *et al*. ^12^	2016	Case-control	SP-RH versus SP-LH	125

LAVH, laparoscopically assisted vaginal hysterectomy; LH, laparoscopic hysterectomy; RCT, randomized clinical trial; RH, robotic-assisted hysterectomy; SP, single-port; VH, vaginal hysterectomy.

### Vaginal hysterectomy

Four studies evaluated VH and compared it with LH ^3, 5, 7^ or LAVH ^5, 8^ ( Table 2).

**Table 2.** Surgical outcomes of vaginal hysterectomy (VH) compared with laparoscopic hysterectomy (LH) or laparoscopically assisted vaginal hysterectomy (LAVH).

Characteristic	Sesti *et al*. ^5^			Lönnerfors *et al*. ^3^		Bogani *et al*. ^7^		Kovachev *et al*. ^8^	
	VH	LH	LAVH	VH	LH	VH	LH	VH	LAVH
Study design	RCT			RCT		Prospective cohort		Prospective cohort	
Number	36	36	36	25	36	40	40	58	129
Uterine weight, g	319.2 ± 107	309.1 ± 88	318.9 ± 100	152 (30–433)	163 (31–694)	80 (30–400)	100 (30–700)	234 ± 68	227 ± 116.5
Pain, n (%)									
None	17 (47)	19 (53)	5 (14)	N/A	N/A	N/A	N/A	N/A	N/A
Mild	10 (28)	11 (30)	22 (62)						
Moderate	4 (11)	2 (6)	4 (11)						
Severe	2 (6)	3 (8)	4 (11)						
Very severe	3 (8)	1 (3)	1 (3)						
Operation time, min	70 ± 19 ^a^	151 ± 4 ^a^	129.6 ± 47 ^a^	59 (29–118)	104 (54–223)	60 (30–140)	75 (20–305)	68 ± 11.1 ^b^	126 ± 18.2 ^b^
Blood loss, mL	182.8 ± 53 ^a^	204 ± 168 ^a^	358.3 ± 67 ^a^	50 ^b^	100 ^b^	100 ^b^	50 ^b^	16 ± 7 ^b, c^	10 ± 5 ^b, c^
Minor complications, n (%)	0	0	0	0	0	N/A	N/A	N/A	N/A
Major complications, n (%)	0	1 (2.8)	2 (5.5)	5 (20)	7 (19.4)	3 (7)	1 (2)	N/A	N/A
Conversion, n (%)	0	0	0	1	2	1 (2)	1 (2)	N/A	N/A
Time of discharge, days	2.1 ± 1 ^a^	3.2 ± 1.2 ^a^	3.2 ± 1.4 ^a^	N/A	N/A	2.4 ± 1.2 ^b^	1.7 ± 1.0 ^b^	N/A	N/A
Concomitant procedures, n (%)	N/A	N/A	N/A	4 (19)	27 (75)	N/A	N/A	N/A	N/A

Pain score evaluated by visual analog scale (VAS) 24 hours post-operatively and was classified as absence of pain (VAS = 0), mild pain (VAS = 1–25), moderate pain (VAS = 26–50), severe pain (VAS = 51–75), and very severe pain (VAS = 76–100). Data are expressed as mean ± standard deviation or mean (range). ^a^ *P*<0.001. ^b^ *P*<0.05. ^c^Drop of hemoglobin concentration (in grams per liter). N/A, data not available; RCT, randomized clinical trial.

VH and LH were compared in a total of 213 patients. Operation time was similar in two studies ^3, 7^ (59–60 versus 75–104 min) and was significantly lower for VH in one of them ^5^ (70 versus 151 min; *P*<0.001). No conversion to laparotomy was observed. Blood loss was significantly lower in the VH group than the LH group in two studies ^3, 5^ (50.0–100 versus 182.8–204.0 mL) and significantly higher in one study ^7^ (100 versus 50 mL; *P*<0.05).

One study ^5^ compared pain measured 24 hours post-operatively by using VAS between VH and LH. Pain score was classified as absence of pain (VAS = 0), mild pain (VAS = 1–25), moderate pain (VAS = 26–50), severe pain (VAS = 51–75), and very severe pain (VAS = 76–100). No difference was observed between VH and LH groups in pain intensity: none (47 versus 53%), mild (28 versus 30%), moderate (11 versus 6%), severe (6 versus 8%), and very severe (8 versus 3%). There was no difference in rates of major complications (vaginal cuff hematoma, vaginal cuff dehiscence, blood transfusion, port infection, re-admission, or re-operation) between VH and LH groups (8/101, 7.9% versus 9/112, 8.0%). No minor complications were reported. Time of discharge was heterogeneous between studies: Sesti *et al*. ^5^ reported faster discharge in the VH group (2.1 ± 1 versus 3.2 ± 1.2 days; *P*<0.001), whereas Bogani *et al*. ^7^ reported faster discharge in the LH group (2.4 ± 1.2 versus 1.7 ± 1.0 days; *P*<0.05).

Two authors ^5, 8^ compared a total of 159 patients submitted to VH or LAVH ( Table 2). Results showed that VH was superior to LAVH in operation time and duration of hospital stay and had shorter duration of procedure and faster hospital discharge. No difference was observed in post-operative pain symptoms after 24 hours ^5^. Blood loss differed between studies: Sesti *et al*. ^5^ showed a greater blood loss in LAVH compared with VH, whereas Kovachev *et al*. ^8^ showed a greater blood loss in VH.

### Single-port laparoscopic hysterectomy

Four studies ^9– 12^ with a total of 505 patients evaluated single-port laparoscopic hysterectomy (SP-LH) ( Table 3 and Table 4). Compared with conventional multi-portal LH or LAVH, SP-LH showed no difference in blood loss, conversion, or minor or major complications. Post-operative pain was variable between studies: two of them ^10, 11^ showed less pain at 18 to 24 hours after surgery in the SP-LH group (2.3–3.0 versus 3.9–4.0; *P*<0.05), whereas one author ^9^ showed no difference (3.0 versus 3.0). Furthermore, Angioni *et al*. ^11^ showed an increase in operative time (89.6 versus 67.3 min; *P*<0.05) in SP-LH but less post-operative pain and shorter length of hospital stay when compared with LH. However, these results were not observed by other authors ^9, 10^.

**Table 3.** Surgical outcomes of single-port laparoscopic hysterectomy (SP-LH) compared with conventional laparoscopic hysterectomy (LH) or conventional laparoscopically assisted vaginal hysterectomy (LAVH).

Characteristic	Kim *et al*. ^9^		Angioni *et al*. ^11^		Song *et al*. ^10^	
	SP-LH	LH	SP-LH	LH	SP-LH	LAVH
Study design	RCT	Case-control	RCT
Number	122	121	31	30	38	38
Uterine weight, g	280 (65–1,380)	298 (60–750)	259.2 ± 44.7 ^a^	296.8 ± 59.2 ^a^	337.9–150.6	324.7–160.1
Pain, visual analog scale	3 (0–9)	3 (0–9)	2.3 ± 0.5 ^b^	4.0 ± 0.9 ^b^	3.0 ± 0.4 ^b^	3.9 ± 0.8 ^b^
Operation time, min	80	69.5	89.6 ^b^	67.3 ^b^	95.8 ± 20.7	95.8 ± 20.7
Blood loss, mL	100	150	63.8 ± 12.5	56.3 ± 11.4	95.8 ± 20.7	95.8 ± 20.7
Minor complications, n (%)	0	1 (0.8)	0	0	3 (8.0)	0
Major complications, n (%)	6 (4.9)	13 (10.3)	1 (3.2)	1 (3.3)	0	0
Conversion, n (%)	5 (4)	1 (0.8)	0	0	5 (13.0)	0
Time of discharge, days	3 (2–7)	3 (2–13)	2.1 ± 0.3 ^b^	2.6 ± 0.6 ^b^	3 (2–5)	3 (2–5)
Concomitant procedures, n (%)	N/A	N/A	5 (16.1)	26 (86.7)	12 (31.0)	11 (29)

Pain score evaluated by visual analog scale (0–10) 18 to 24 hours after surgery. Data are expressed as mean ± standard deviation or mean (range). ^a^ *P*<0.05. ^b^ *P*<0.001. N/A, not available; RCT, randomized clinical trial.

**Table 4.** Surgical outcomes of single-port robotic hysterectomy (SP-RH) compared with single-port laparoscopic hysterectomy (SP-LH) and robotic hysterectomy (RH) compared with vaginal hysterectomy (VH).

Characteristic	Paek *et al*. ^12^		Lönnerfors *et al*. ^3^	
	SP-RH	SP-LH	RH	VH
Study design	Case-control		RCT	
Number	25	100	61	25
Uterine weight, g	271 ± 119	249 ± 190	180 (54–1,114)	163 (31–694)
Pain, visual analog scale	3 (2–4) ^a^	4 (2–5) ^a^	N/A	N/A
Operation time, minutes	170.9 ± 65.5 ^a^	88.3 ± 38.4 ^a^	76 (43–210)	59 (29–118)
Blood loss, mL	20 ^b^	50 ^b^	50	50
Minor complications, n (%)	0	5 (1.1)	0	0
Major complications, n (%)	0	6 (1.3)	4 (6.6) ^b^	5 (20) ^b^
Time discharge, days	3.5 ± 0.7	3.8 ± 1.4	N/A	N/A
Concomitant procedures	N/A	N/A	27 (75)	36 (59)

Pain score evaluated by visual analog scale (0–10) 12 hours after surgery. Data are expressed as mean ± standard deviation or mean (range). ^a^ *P*<0.001. ^b^ *P*<0.05. N/A, not available; RCT, randomized clinical trial.

### Robotic hysterectomy

Two studies evaluated robotic-assisted laparoscopic hysterectomy for benign pathologies ^3, 12^. Single-port robotic hysterectomy (SP-RH) was compared with SP-LH by Paek *et al*. ^12^ in 2016 with a total of 125 patients. Results showed benefits in post-operative pain (evaluated by VAS), operation time, and blood loss in the SP-LH group compared with the SP-RH group. There was no difference in rates of minor (0% versus 1.1%) or major (0% versus 1.3%) complications between the SP-RH and SP-LH groups. Intra-operative blood loss was superior in the SP-LH group (50 versus 20 mL; *P*<0.05) ( Table 4).

Lönnerfors *et al*. ^3^ included a total of 86 patients submitted to VH or RH ( Table 4). Concomitant procedures were performed in 4 (19.0%) of the patients in the VH group and 36 (59.0%) in the RH group ( *P*=0.47). There was no difference in operation time, blood loss, or conversion to laparotomy between VH or RH. No minor complications were observed. This study reported significantly less major complications (vaginal cuff hematoma, vaginal cuff dehiscence, and port infection) in RH (n=4, 6.6%) compared with VH (n=5, 20.0%; *P*<0.05).

## Discussion

This systematic review evaluated nine studies from the last 3 years, looking to recent advances and surgical outcomes for total hysterectomy using minimally invasive techniques for benign diseases. Our findings reveal that the oldest technique for uterus removal using the vaginal route remains the preferable option for most indications of benign hysterectomy. Alternatively, LH brings some benefits for some specific cases. We observed that in the last 3 years all randomized and prospective trials were related to minimally invasive techniques. None of them included abdominal hysterectomy (by laparotomy) in the analyses, although this type of procedure is still frequently performed worldwide.

In the present review, included studies revealed that VH showed lower operation time and shorter hospital stay compared with LH. Also, although studies have shown different results regarding blood loss during VH compared with LH and LAVH, no significant difference was observed in peri-operative complications with this technique. These results are in agreement with a meta-analysis from 2014 ^1^, showing that for the same uterus size and vaginal access, VH has the same clinical outcomes as LH or LAVH, has faster recovery and less costs. On the other hand, LH and LAVH offer some advantages when compared with VH, such as the possibility of abdominal exploration. Also, they are preferable in patients with suspicion of adhesions, ovarian cysts/mass, and endometriosis ^5^.

Single-port laparoscopic surgeries have been recently introduced in order to maximize minimally invasive advantages by reducing the number of incisions to one. In this review, we found controversial results in terms of post-operative pain and length of hospital stay between SP-LH and conventional LH or LAVH. A previous review showed no difference in return to normal activities, major complications, quality of life, and patient satisfaction in SP-LH compared with LH despite a better cosmetic result ^1^. Furthermore, single-port is a challenging technique since it is made technically difficult by the clash between instruments, instability of the camera, the limited mobility of straight instruments, and the lack of instrument triangulation ^12^.

In this sense, robotic surgery has emerged as a feasible option for single-port surgeries, increasing surgical precision, visualization, and ergonomics ^12^. We found that in comparison with conventional SP-LH, SP-RH had no advantages in operation time but was related to less post-operative pain. No differences in complication rate were observed. Similarly, there were no advantages in operation time, blood loss, or conversion in multi-portal RH compared with VH. These results are in agreement with previous studies, which showed lack of evidence of RH benefits over other conventional techniques for benign uterine conditions but did show greater costs ^1, 12, 13^. However, there was one study, by Lim *et al.* ^14^, that estimated the incidence and reasons for conversion to laparotomy in women submitted to LH for benign gynecologic indications. Overall, almost 7,000 women underwent an attempted LH with 3.93% (n=275) converted to laparotomy. After adjusting for intra-operative associated-factors, hysterectomy indication, and socioeconomic differences, the authors observed a decreased odds of conversion to laparotomy with the use of robotic-assisted laparoscopy compared with conventional laparoscopy (adjusted odds ratio 0.14, 95% confidence interval 0.07–0.25) with a predicted risk of conversion of 0.8% compared with 5.4% ( *P*<0.001) ^14^.

In summary, updated evidence suggests that for hysterectomy in benign conditions VH is still superior to LH, LAVH, and RH regarding hospital stay and operation time and has the same complication rate and lower costs. Pre-operatively, it is important to consider possible contraindications for the vaginal route as discussed in the present review. SP-H performed by either laparoscopy or robotic-assisted laparoscopy emerged as a novel minimally invasive technique, but according to the available data, it currently has no clear advantages compared with VH or conventional LH. This updated information, despite all of the advances in technologies and surgical equipment, brings up advantages of one of the oldest approaches for uterus removal, VH. Limitations of this review include the studies’ heterogeneity, imprecision of results of included studies, and inadequate report of outcomes. (For example, some studies did not include information regarding concomitant procedures and time of discharge; other studies did not report outcomes on pain using the VAS, making it impossible to compare them with studies that performed this evaluation.) Certainly, more prospective studies, preferably randomized clinical trials, are still required to compare all aspects of the different routes for benign hysterectomy, including new advances and technologies such as SP-RH.
